# Inflammatory Disturbances in Preeclampsia: Relationship between Maternal and Umbilical Cord Blood

**DOI:** 10.1155/2012/684384

**Published:** 2012-05-23

**Authors:** Cristina Catarino, Alice Santos-Silva, Luís Belo, Petronila Rocha-Pereira, Susana Rocha, Belmiro Patrício, Alexandre Quintanilha, Irene Rebelo

**Affiliations:** ^1^Serviço de Bioquímica, Faculdade de Farmácia da Universidade do Porto (FFUP), 4050-313 Porto, Portugal; ^2^Instituto de Biologia Molecular e Celular (IBMC), Universidade do Porto, 4150-180 Porto, Portugal; ^3^Centro de Investigação em Ciências da Saúde (CICS), Universidade da Beira Interior, 6201-506 Covilhã, Portugal; ^4^Serviço de Obstetrícia e Ginecologia, Hospital de S. João, 4200-319 Porto, Portugal; ^5^Faculdade de Medicina, Universidade do Porto, 4200-319 Porto, Portugal; ^6^Instituto de Ciências Biomédicas Abel Salazar (ICBAS), Universidade do Porto, 4050-313 Porto, Portugal

## Abstract

Preeclampsia (PE) is one of the main causes of maternal and fetal mortality and morbidity. PE is associated with an inflammatory state and with oxidative stress, in maternal circulation. Our aim was to evaluate and compare the levels of oxidative stress and inflammatory markers in maternal and umbilical cord blood (UCB), in normal and PE pregnancies. We measured acute-phase proteins (CRP and *α*1-antitrypsin), proinflammatory cytokines (IL-6 and TNF-*α*), leukocyte activation (elastase, lactoferrin, sL-selectin, sVCAM, sPECAM), total antioxidant status (TAS), thiobarbituric acid reactive substances (TBARS), and uric acid levels. We studied 42 healthy pregnant women, 46 PE women, and their neonates. The concentrations of IL-6, TNF-*α*, *α*1-antitrypsin, CRP, sVCAM, uric acid, and TBARS were significantly higher, and sL-selectin was significantly lower in PE pregnant women as compared with normotensive pregnant women. In newborns uric acid, *α*1-antitrypsin, and CRP values were significantly higher in PE; leukocyte count, sL-selectin, lactoferrin, and the ratio elastase/*α*1-antitrypsin were significantly lower. Our data suggest that PE pregnancy is associated with an enhanced maternal inflammatory condition, which is reflected in fetal circulation. This enhanced inflammatory state seems to be related to endothelial dysfunction and increased cytokine synthesis, rather than with neutrophil activation.

## 1. Introduction

Preeclampsia (PE) is a human specific pregnancy and multisystem disorder, which may cause maternal and neonatal morbidity and mortality. The specific cause for this syndrome remains unclear, despite the intense investigation. It has been reported that an altered lipid profile [1], leukocyte activation [2], enhanced inflammatory response [3], and oxidative stress [4], in maternal circulation, are frequently associated with development of this disorder. In PE, the hypoperfused placenta is a potential source of reactive oxygen species (ROS) and cytokines [5, 6], which may induce oxidative stress and endothelial cell dysfunction, as well as an inflammatory response, in the mother.

An inflammatory response is usually accompanied by increasing concentrations of proinflammatory cytokines, acute-phase proteins and may involve leukocyte activation [7]. Tumor necrosis factor-alpha (TNF-alpha) and IL-6 are some of the pro-inflammatory cytokines that seem to play a role in immune activation in PE [8]. C-reactive protein (CRP) and alpha-1-antitrypsin increase rapidly in response to an inflammatory stimulus [9]. An increase in circulating leukocytes is usually observed in inflammation, and cell adhesion molecules (CAM) are crucial for the interaction between leukocytes and endothelial cells. L-Selectin is a glycoprotein expressed by almost all leukocytes, which is responsible for leukocyte adhesion to the endothelium and rolling along blood vessels [10]. With leukocyte activation, sL-selectin is released from the surface of these cells, due proteolytic cleavage. Vascular cell adhesion molecule 1 (VCAM-1), a member of the Ig family, induces a firm leukocyte adhesion to the endothelial cells, and is also released by proteolytic cleavage (sVCAM-1). Platelet endothelial cell adhesion molecule 1 (PECAM-1) plays an important role in transmigration of leukocytes across the endothelial layer [10].

The activation of neutrophils may occur in the presence of some cytokines (i.e., TNF-*α*) and of some chemoattractants released during an inflammatory process. During neutrophil activation, there is a metabolic activation and release of their granules in blood and in tissues, contributing to increase the inflammatory response and/or oxidative stress [11]. Elastase and lactoferrin are two compounds frequently measured in plasma to access neutrophil activation in vivo [12]. Elastase, stored in the azurophilic granules of neutrophils, has different biological effects, such as proteolytic activity and bactericidal properties; it is also involved in cytokine induction, platelet and lymphocyte activation [13]. Lactoferrin is a glycoprotein stored in the specific or secondary granules of the neutrophils [12]; it is involved in the regulation of the immune response and is associated to an antimicrobial activity and to an anti-inflammatory effect. The protective action of lactoferrin is due to the reduction in the synthesis of pro-inflammatory cytokines, namely, TNF-*α* and IL-6 [14].

PE often associated with premature delivery and increased risk for delivering newborns small for their gestational age (SGA). Few studies focused their attention on fetal circulation, in the presence of this syndrome. In a previous study, we showed that PE pregnancy is associated with changes in endothelial function in fetal circulation [15]. In the present study, we searched for a possible relation between maternal and fetal inflammatory modifications, in both normal and PE pregnancies.

The aim of this study was to evaluate and compare oxidative stress and inflammatory markers in maternal and umbilical cord blood (UCB) in normal and PE pregnancies. We measured some inflammatory markers, namely, acute phase proteins (CRP and *α*1-antitrypsin), pro-inflammatory cytokines (IL-6 and TNF-*α*) and leukocyte activation markers (elastase, lactoferrin, sL-selectin, sVCAM, sPECAM). To evaluate oxidative stress, we measured total antioxidant status (TAS), thiobarbituric acid reactive substances (TBARS) and uric acid levels.

## 2. Methods

### 2.1. Subjects

This study was performed under approval of the Ethics Committee of the Hospital S. João, Porto, Portugal, and every enrolled pregnant woman gave their informed consent to participate in the study; 42 healthy pregnant women, 46 PE pregnant women, and their neonates were included in the study. Clinical and ultrasound evaluations were used in the diagnoses of normal pregnant women, and all of them presented a normal course and outcome of pregnancy. All patients with any history of pregestational diabetes, gestational diabetes *mellitus*, cardiovascular disease, renal and liver diseases, as well as in the case of fetal anomalies and maternal or fetal infection, were excluded from the study.

PE was defined according to the International Society for the Study of Hypertension in Pregnancy classification (diastolic blood pressure higher than 110 mmHg on one occasion, or exceeding 90 mmHg on repeated measures; proteinuria of ≥0.3 g/24 h, or ≥1+ proteinuria on dipstick testing on repeated measures, after the 20th week of gestation) [16].

According to Portuguese guidelines [17], newborns were defined as small for gestational age (SGA) when presenting a birth weight below the 10th percentile for gestational age, as adequate for gestational age (AGA) when presenting a birth weight above the 10th percentile and below the 90th percentile for gestational age, and as large for gestational age (LGA) when presenting a birth weight above the 90th percentile for gestational age. Apgar scores at 1 and 5 minutes were evaluated by the health caregivers responsible for immediate neonatal support.

It was very difficult to match normal and PE women for gestational age, as a normal pregnancy does not associate a premature delivery, as often occur in PE pregnancies. Considering the differences in gestational age presented by normal and PE cases, an adjustment for gestational age was performed for all sets of data.

### 2.2. Collection and Handling of Blood Samples

Maternal blood was collected from an antecubital vein before delivery. UCB was collected immediately after delivery of the placenta. Blood samples were processed within 2 hours after collection. Whole blood (EDTA as anticoagulant) was used to perform hematologic studies. Blood serum and plasma were separated from blood cells and aliquots were stored at −70°C until assayed.

### 2.3. Biochemical Analysis

Commercially available specific sandwich enzyme-linked immunosorbent assay (ELISA) kits were used to evaluate plasma levels of IL-6, TNF-*α*, sL-selectin (R&D Systems, Minneapolis, MN, USA), sICAM, sPECAM (IBL Immuno-Biological Laboratories, Hamburg), lactoferrin (Bioxytech Lactof EIA, Oxis Research, Foster City, CA, USA), and elastase (Human PMN elastase ELISA, Bender MedSystems, Vienna, Austria). CRP was determined in serum samples by using a high-sensitivity ELISA technique, as described elsewhere [18]. *α*1-antitrypsin, uric acid, and TAS concentrations were measured by routine procedures, using commercially available kits (*α*1-antitrypsin, Roche, Basel, Switzerland; uric acid and Total Antioxidant Status, Randox Laboratories, UK).

Leukocyte count was performed by using an automatic blood cell counter (ABX Micros 60-OT, Horiva-ABX, France). Leukocyte differential count was evaluated in Wright stained blood films.

TBARS was evaluated according to the method of Niehaus and Samuelsson described elsewhere [19].

### 2.4. Statistical Analysis

Measurements are presented as mean ± standard deviation or as median (interquartile range). For statistical analysis, we used the Statistical Package for Social Sciences (SPSS, version 17.0, SPSS Inc, Chicago) for Windows. To compare the studied groups, we used the Student's unpaired *t*-test, whenever parameters presented a Gaussian distribution, and the Mann-Whitney *U*-test in the case of a non-Gaussian distribution. The number of SGA, AGA, and LGA cases and Apgar score lower than 7 were compared between normal and preeclamptic pregnancy by Chi-square test. Adjustment of statistical differences for gestational age was performed by analysis of covariance (ANCOVA). Spearman's rank correlation coefficient was used to evaluate relationships between sets of data. A *P* value lower than 0.05 was considered as statistically significant.

## 3. Results

### 3.1. Patient's Clinical Data

Clinical data from normal and PE pregnant women, and from their neonates, are documented in Table 1. Proteinuria and high blood pressure were characteristic of PE patients. Fetal weight, placental weight, and gestational age were significantly lower in PE cases, and the number of cases with Apgar ≤7 at 1 minute was significantly higher in PE cases, as compared to normal cases; SGA newborns were observed only in the PE group. The two groups presented similar values for maternal age and body mass index (BMI). Considering the observed differences in gestational age, we further adjusted our maternal and fetal data for gestational age. First, we present the results observed for the total sets of data and, afterwards, the results obtained after adjustment for gestational age.

### 3.2. Maternal Blood

In Table 2, cytokines, acute-phase proteins, and oxidative stress markers are presented for the studied groups, in normal and PE cases. We found significantly higher values for uric acid and TBARS in PE group and similar values for TAS. IL-6, TNF-*α*, *α*1-antitrypsin, and CRP concentrations were significantly higher in PE pregnant women when compared with normotensive pregnant women.

We also found significantly higher values for basophil count and sVCAM levels, and a trend to higher leukocyte count in PE (Table 3). Soluble L-selectin concentrations were significantly lower in PE pregnant women. No statistical differences were observed in total leukocyte, neutrophil, eosinophil, lymphocyte and monocyte counts, sPECAM, elastase, and lactoferrin levels between the groups.

### 3.3. Umbilical Cord Blood

When comparing UCB data from normal and preeclamptic pregnancies (Table 2), we found similar values for IL-6, TNF-*α*, TAS, and TBARS; however, significantly higher uric acid, *α*1-antitrypsin and CRP values were observed in PE.

Similar values were found for basophil count, sPECAM, and the ratios of elastase/neutrophil, lactoferrin/neutrophil in UCB from neonates of PE mothers. However, the newborns from the PE group presented significantly lower values for total leukocyte, neutrophil, eosinophil, lymphocyte and monocyte counts, sL-selectin, lactoferrin, and the ratio of elastase/*α*1-antitrypsin (Table 3).

We adjusted both maternal and UCB data for gestational age and after that, we still observed the same differences, suggesting that the differences observed were not significantly affected by gestational age.

As both normal and preeclamptic groups included about 25% of labouring cases (as referred in Table 1), we compared eutocic versus dystocic deliveries and no differences were found between groups.

### 3.4. Correlations

**Figure 1:**
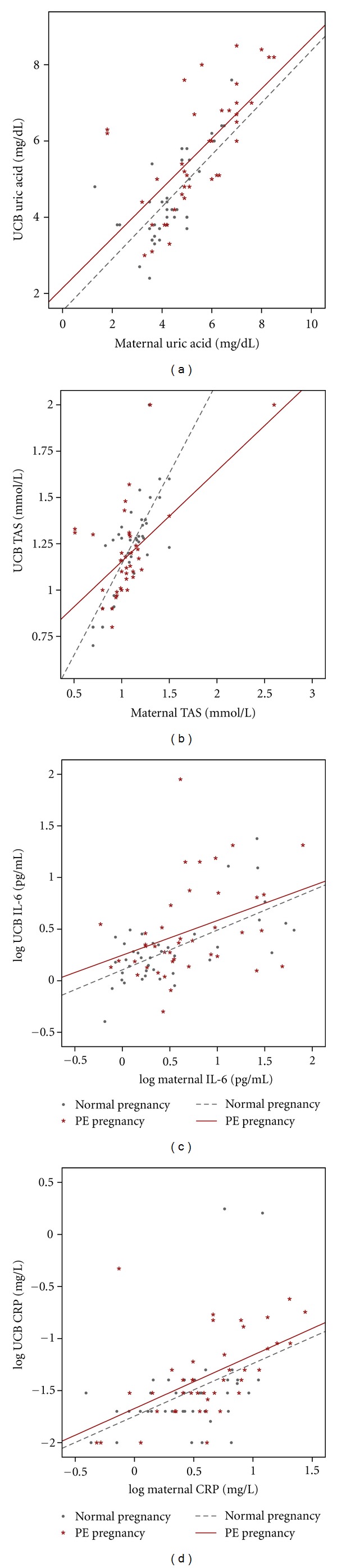
Correlation between maternal and UCB levels of uric acid (a), TAS (b), IL-6 (c), and CRP (d), in normal and PE pregnancies.

We found significant positive correlations between maternal and UCB uric acid (Figure 1(a)), in normal pregnancy (Pearson correlation coefficient, 0.70; *P* < 0.001) and PE pregnancy (Pearson correlation coefficient, 0.68; *P* < 0.001); a significant positive correlation between maternal and UCB TAS (Figure 1(b)), in normal pregnancy (Pearson correlation coefficient, 0.79; *P* < 0.001) and PE pregnancy (Pearson correlation coefficient, 0.58; *P* < 0.001); a significant positive correlation between UCB and maternal IL-6 (Figure 1(c)) in normal pregnancy (Spearman's correlation coefficient, 0.55; *P* < 0.001) and PE pregnancy (Spearman's correlation coefficient, 0.44; *P* = 0.004), and a significant positive correlation between maternal CRP and UCB CRP (Figure 1(d)), in normal pregnancy (Spearman's correlation coefficient, 0.43; *P* = 0.007) and PE pregnancy (Spearman's correlation coefficient, 0.61; *P* < 0.001).

**Figure 2:**
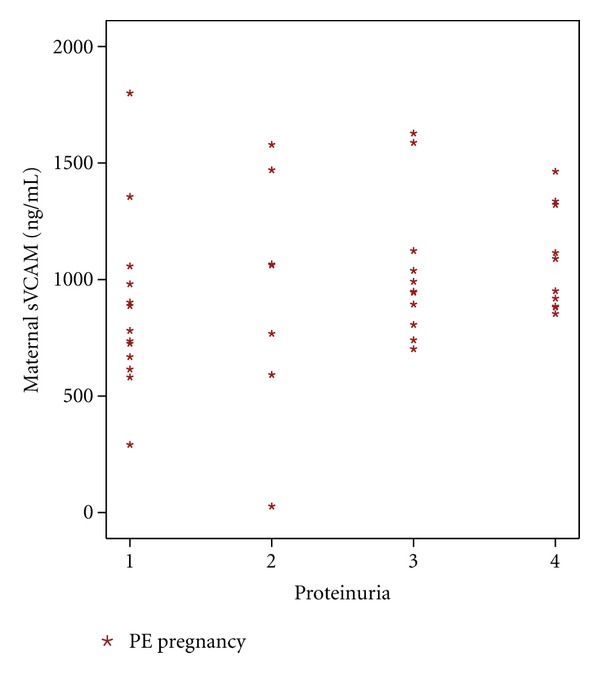
Correlation between maternal proteinuria and sVCAM, in PE (Spearman's correlation coefficient, 0.33; *P* = 0.027).

**Figure 3:**
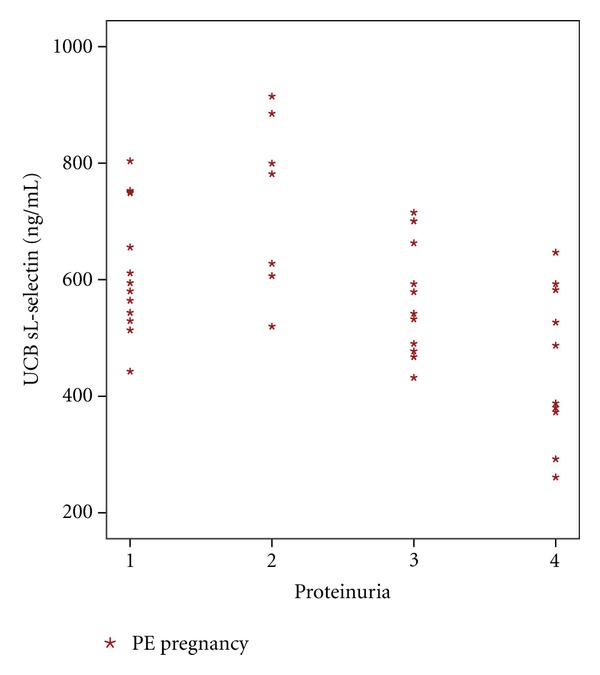
Correlation between maternal proteinuria and UCB sL-selectin, in PE (Spearman's correlation coefficient, −0.45; *P* = 0.003).

Maternal concentrations of sVCAM and proteinuria were positively correlated in PE (Spearman's correlation coefficient, 0.33; *P* = 0.027) (Figure 2); UCB sL-selectin levels correlated negatively with maternal proteinuria, in PE (Spearman's correlation coefficient, −0.45; *P* = 0.003) (Figure 3).

## 4. Discussion and Conclusions

In this study, we confirmed an enhanced inflammatory process in maternal circulation, in PE. We observed in PE women, when compared with normal pregnant women, significantly higher levels for acute-phase proteins, namely, CRP and *α*1-antitrypsin, and for the proinflammatory cytokines, IL-6 and TNF-*α* (Table 2). The higher CRP concentrations observed in the PE group are consistent with previous reports [20–23]. Moreover, Thilaganathan et al. [22] reported that women, who subsequently developed PE, presented significantly higher levels for CRP at a lower gestational age (16 weeks). To our knowledge, this is the first study evaluating *α*1-antitrypsin in PE. We found significantly higher values in PE women, which are in accordance with the enhanced acute-phase response observed in PE, as suggested by CRP.

The increase in the CRP levels is accompanied by an increase in pro-inflammatory cytokines, in PE maternal circulation. The increase in IL-6 and TNF-*α* levels in PE women is in accordance with some previous studies [4, 24]; however, other authors reported no differences between normal and PE pregnancy for IL-6 [25, 26] and for TNF-*α* [27]. Conflicting results also exist concerning cytokine placental production [28–30]. Most of these placental studies suggest that placenta becomes a considerable source of cytokines along pregnancy that is disturbed in PE and may contribute to the higher levels of those cytokines, in maternal circulation.

There is no doubt about the involvement of an inflammatory process in the PE physiopathology; nevertheless, there is no agreement about the role of leukocyte activation in PE [2, 31]. Several studies reported that PE women present an increased number of total leukocytes and a higher neutrophil count [32]. Matsuo et al. [33] showed that total leukocyte count and neutrophil number were higher in PE patients, as compared with normotensive women, before the symptomatic phase of PE. In our study, no significant differences were found in leukocyte and neutrophil counts; nevertheless, we observed a trend towards higher values in the PE group (Table 3). Basophil count was significantly higher in PE but this is not a consistent finding in the literature [34–36].

Contradictory findings have been reported for cellular adhesion molecules, in placenta and in maternal circulation, in PE. Tziotis et al. [37] reported similar values for placental expression for PECAM-1, ICAM-1, VCAM-1, in normal and PE pregnancies. In the present study, we found in maternal blood, in PE, significantly higher sVCAM concentrations, significantly lower sL-selectin concentrations and similar values for sPECAM. These results are in agreement with Chaiworapongsa et al. [38]; however, controversial results have been published [27, 39–41].

The increase in sVCAM may reflect leukocyte adhesion to the endothelium or an endothelial dysfunction. The results concerning sL-selectin in PE are still controversial [2, 42]. Some authors proposed that under an acute inflammatory condition, sL-selectin rises [43], while under a chronic inflammatory condition, a decrease in circulating sL-selectin levels occurs [44]. The decrease in sL-selectin may be a result of downregulation of leukocyte L-selectin expression [44], which might reflect a mechanism to counteract the enhanced inflammatory process observed in PE. Another possible explanation is that leukocyte L-selectin expression is accompanied by an opposite change in the soluble form [45, 46], thus, our results (sL-selectin decreased levels) may indeed reflect an upregulation of leukocyte L-selectin expression, improving leukocyte-endothelial interaction.

The increase in sVCAM and the decrease in sL-selectin, in PE, suggest leukocyte adhesion to endothelial cells, even though the similar sPECAM levels suggest that leukocyte tissue transmigration is unlikely to occur.

In a previous study [15], we described that PE women presented significantly higher tPA and PAI-1 values, when compared with normal pregnant women. Both tPA and PAI-1, as well as sVCAM, are produced by endothelial cells. In the same study we also described that D-dimer did not change, though it was expected to be higher, in accordance with the increase in tPA, which is an activator of the fibrinolytic system. All data suggest an endothelial dysfunction that might also explain the rise observed in sVCAM, in PE women. Moreover, the severity of the disease might be related with endothelial dysfunction. Indeed, we found a significant positive correlation between sVCAM and proteinuria (an accepted marker of severity of PE) (Figure 2), and we also found the same type of correlation between tPA and PAI-1, with proteinuria, in PE women [15].

Concerning markers of neutrophil degranulation, we studied elastase and lactoferrin, but no differences were found between normal and PE pregnant women (Table 3). Few studies measured elastase and lactoferrin levels in maternal circulation in PE and data are not consistent [47, 48].

At first view, we may think that elevated *α*1-antitrypsin levels could mask the unchanged elastase levels, since *α*1-antitrypsin is involved in elastase inhibition, but similar values were found between the studied groups after the calculation of elastase/*α*1-antitrypsin ratio. Similar results, between normal and PE pregnant women, for elastase and lactoferrin levels and in the ratios of elastase/neutrophil, lactoferrin/neutrophil, and elastase/*α*1-antitrypsin, seem to be indicative that PE is not associated with neutrophil degranulation. Our results contradict previous studies, which showed an association between PE and neutrophil degranulation; Mellembakken et al. [49] showed an increase in myeloperoxidase; Belo et al. [20], Gupta et al. [47], and Lok et al. [2] observed higher elastase levels in PE; nevertheless, no differences were found for lactoferrin concentration [20, 49].

The contribution of oxidative stress to PE has been accepted, although the exact changes in antioxidants/oxidants are still controversial [4, 6, 50, 51]. In our study, we found significantly higher levels for TBARS in PE pregnant women, when compared with normal pregnancy, but no differences were detected in TAS (Table 2). TBARS are a biomarker of lipid peroxidation, and the increased levels in maternal circulation are in accordance with the contribution of oxidative stress to PE. TAS is used as an indicator of the whole antioxidant capacity, thus, different molecules with antioxidant properties may contribute to TAS, such as albumin, uric acid, bilirubin, vitamin C, vitamin E, and bioflavonoids [52].

Higher levels of uric acid are a common characteristic of PE patients and may contribute to the pathogenesis of the disorder [53]. Antioxidant properties have been also attributed to uric acid [54]. In our study, uric acid levels were significantly higher in PE and are likely to contribute to TAS levels. Furthermore, the TBARS/TAS ratio is significantly elevated in PE women, and it is quite possible that this imbalance could be caused by an enhanced oxidative stress or a decrease in specific antioxidant compounds, or even a contribution of both.

Few data exist concerning inflammation in UCB in the presence of PE; the present study was performed in order to analyze if the modifications observed in maternal circulation in PE were also observed in UCB. We found evidence of increased inflammation in fetal circulation, in the presence of PE. We observed, in cord blood from PE pregnant women, a significant increase in CRP and *α*1-antitrypsin levels, a trend towards higher values of IL-6 and no differences in TNF-*α*. Contrary to our results, Braekke et al. [55] reported no differences, between newborns from PE and normotensive pregnancies, regarding calprotectin and CRP levels.

This inflammatory profile in UCB from PE cases was also associated, as occurred in the mothers, with a significant reduction in sL-selectin and a significant rise in sVCAM, suggesting that a similar mechanism might underlie those changes.

Total leukocyte number was significantly lower in cord blood from PE pregnancy, as well as neutrophil count. This finding seems to be a contrasense, as usually inflammation is characterized by leukocytosis and neutrophilia. Tsao et al. [56] demonstrated that pregnancy-induced hypertension is associated with low neutrophil count and granulocytes colony stimulating factor (G-CSF) levels in preterm newborns. We did not measure G-CSF in our samples, but, in fact, the decrease in the G-CSF may contribute to this leucopenia, as G-CSF is an important hematopoietic growth factor for granulocyte differentiation and proliferation [57]. Strengthening this hypothesis, a lower number of cases presenting neutrophilic myelocytes and metamyelocytes were identified in PE. We may also hypothesize that these leukocyte changes might include the physiologic response to counteract the enhanced inflammation observed.

Different expressions of adhesion molecules on neutrophils, namely, integrins, selectins, and Ig superfamily, were shown to be upregulated in infants from PE pregnancies [46, 58] suggesting an interaction between neutrophils and endothelial cells. Nevertheless, Mellembakken et al. [46] also reported, in the same samples, that plasma levels of soluble L-selectin were significantly decreased in PE infants.

No published data were found concerning plasma levels of products released from neutrophil granules in fetal circulation. In the present study, PE infants presented significantly lower values for elastase and lactoferrin; after calculation of elastase/neutrophil and lactoferrin/neutrophil, statistical significance was lost. These findings suggest that, as in the mother, neutrophil degranulation does not seem to occur in fetal circulation.

Conflicting results on oxidative stress in PE fetal circulation are published [59–61]. In the present study, similar values for TAS and TBARS were found between newborns from normal and PE pregnancies. Nevertheless, infants born from PE pregnancy presented significantly higher uric acid concentration, which may contribute to TAS levels in PE, as we observed in maternal blood.

Considering the changes observed in normal and PE cases (in maternal and cord blood), we studied some correlations between maternal and UCB values, to establish a possible relationship between them. Increased maternal concentration of CRP and IL-6, in PE condition, was reflected in the fetal circulation, since positive correlations were found between maternal and UCB for both CRP and IL-6.

In summary, our data suggest that PE pregnancy is associated with an enhanced maternal inflammatory condition, also reflected in fetal circulation. This enhanced inflammatory state seems to be related to endothelial dysfunction and increased cytokine synthesis rather than with neutrophil activation.

## Figures and Tables

**Table 1:** Clinical data of normal and PE cases at delivery.

	Control pregnancies (*n* = 42)	PE pregnancies (*n* = 46)	*P *value
Age (years)	30.4 ± 5.7	29.7 ± 5.3	0.52
Blood Pressure (mm Hg):			
Systolic	119.9 ± 11.5	155.1 ± 14.6	<0.001
Diastolic	69.0 ± 7.2	97.1 ± 6.3	<0.001
Cases presenting proteinuria [*n* (%)]			
Cases with 1+	—	16 (34.8%)	
Cases with 2+	—	7 (15.2%)	
Cases with 3+	—	13 (28.7%)	
Cases with 4+	—	10 (21.7%)	
Gestational age (weeks)	38.5 (38.0; 39.3)	37.0 (34.0; 38.0)	<0.001
Body Mass Index (kg/m^2^)	29.2 (27.2; 30.8)	29.8 (26.8; 32.8)	0.16
Placental weight (g)	626.0 (542.5; 779.8)	510.0 (392.5; 560.0)	<0.001
Birth weight (kg)	3.4 (3.0; 3.7)	2.6 (1.8; 3.0)	<0.001
Apgar Score ≤ 7 [*n* (%)]:			
1 min	1 (2.4%)	7 (15.2%)	0.04
5 min	0 (0%)	0 (0%)	1.0
SGA/AGA/LGA (*n*)	0/42/0	7/39/0	0.008
Cesarean section [*n* (%)]	31 (73.8%)	36 (78.3%)	0.63

*n*, number of cases; PE, preeclampsia; SGA, small for gestational age; AGA, adequate for gestational age; LGA, large for gestational age.

**Table 2:** Oxidative stress parameters, cytokines, and acute-phase proteins in maternal and cord blood, in normal and PE cases.

	Maternal			UCB		
	Normal (*n* = 42)	PE (*n* = 46)	*P *Value	Normal (*n* = 40)	PE (*n* = 42)	*P *Value
Uric acid (mg/dL)	4.2 ± 1.3	5.5 ± 1.6	<0.001	4.5 ± 1.1 (*n* = 39)	5.7 ± 1.6 (*n* = 40)	<0.001
TAS (mmol/L)	1.10 ± 0.22	1.07 ± 0.3	0.55	1.23 ± 0.27 (*n* = 39)	1.17 ± 0.26 (*n* = 41)	0.143
TBARS (*μ*mol/L)	1.50 (1.29; 1.81) (*n* = 40)	1.87 (1.49; 2.38) (*n* = 43)	0.013	1.08 (0.95; 1.26) (*n* = 35)	1.10 (0.85; 1.36) (*n* = 35)	0.99
TBARS/TAS	1.48 (1.19; 1.66) (*n* = 40)	1.76 (1.46; 2.13) (*n* = 43)	0.003	0.93 (0.76; 1.31) (*n* = 35)	0.93 (0.71; 1.25) (*n* = 35)	0.84
IL-6 (pg/mL)	1.9 (1.2; 6.4)	4.0 (2.3; 10.1) (*n* = 42)	0.013	1.8 (1.2; 2.8)	2.2 (1.5; 5.6)	0.066
TNF-*α* (pg/mL)	1.2 (1.0; 1.6)	1.9 (1.3; 2.4) (*n* = 43)	0.001	1.9 (1.4; 2.4)	1.8 (1.5; 2.1)	0.34
*α*1-Antitrypsin (mg/dL)	191.6 ± 57.0	250.5 ± 74.9	<0.001	120.2 ± 30.1 (*n* = 39)	134.1 ± 23.5 (*n* = 40)	0.025
CRP (mg/L)	3.1 (1.4; 5.8)	4.1 (2.5; 7.9)	0.033	0.03 (0.02; 0.04) (*n* = 39)	0.04 (0.02; 0.08)	0.011

*n*, number of cases; PE, Preeclampsia; UCB, umbilical cord blood.

**Table 3:** Leukocyte activation parameters in maternal and umbilical cord blood, in normal and PE cases.

	Maternal			UCB		
	Normal (*n* = 42)	PE (*n* = 46)	*P *Value	Normal (*n* = 39)	PE (*n* = 42)	*P *Value
Leukocytes (×10^9^/L)	10.8 ± 3.9	11.90 ± 3.74	0.19	12.2 ± 3.5 (*n* = 41)	9.6 ± 4.8 (*n* = 46)	0.006
Neutrophils (×10^9^/L)	8.4 ± 3.7	9.17 ± 3.74	0.32	5.7 ± 2.1 (*n* = 40)	4.04 ± 2.8 (*n* = 46)	0.002
Eosinophils (× 10^9^/L)	0.07 ± 0.09	0.056 ± 0.093	0.38	0.5 ± 0.41 (*n* = 40)	0.32 ± 0.35 (*n* = 46)	0.033
Basophils (×10^9^/L)	0.007 ± 0.026	0.025 ± 0.039	0.01	0.06 ± 0.077	0.035 ± 0.064	0.073
Lymphocytes (×10^9^/L)	1.9 ± 0.6	2.22 ± 1.06	0.10	5.19 ± 1.37	4.46 ± 1.82	0.044
Monocytes (×10^9^/L)	0.4 ± 0.3	0.39 ± 0.21	0.89	0.86 ± 0.51	0.65 ± 0.44	0.039
Metamyelocytes (*n*)	10	6	0.27	19	17	0.38
Myelocytes (*n*)	3	1	0.34	10	5	0.15
sVCAM (ng/mL)	816.1 ± 200.9 (*n* = 38)	948.6 ± 360.4 (*n* = 45)	0.04	2581.6 ± 1081.6 (*n* = 34)	3101.4 ± 1090.8 (*n* = 39)	0.045
sPECAM (ng/mL)	146.9 ± 25.5 (*n* = 39)	142.8 ± 28.3 (*n* = 45)	0.49	114.0 ± 17.3 (*n* = 35)	108.7 ± 18.7 (*n* = 37)	0.22
sL-selectin (ng/mL)	640.6 ± 119.6 (*n* = 38)	564.2 ± 119.1 (*n* = 42)	0.005	697.8 ± 127.5 (*n* = 38)	587.0 ± 148.3 (*n* = 42)	0.001
Elastase (ng/mL)	52.0 (39.9; 64.8) (*n* = 38)	60.8 (47.6; 75.9) (*n* = 41)	0.16	41.3 (27.8; 54.7) (*n* = 37)	28.4 (20.7; 41.4) (*n* = 41)	0.013
Elastase/Neutrophil (pg)	6.7 (5.5; 8.3) (*n* = 38)	7.2 (4.7; 9.4) (*n* = 41)	0.9	6.2 (5.1; 9.9) (*n* = 37)	8.5 (5.3; 12.3) (*n* = 41)	0.09
Elastase/*α*1-Antitrypsin (×10^−4^)	0.27 (0.20; 0.43) (*n* = 38)	0.25 (0.18; 0.32) (*n* = 41)	0.22	0.33 (0.23; 0.46) (*n* = 35)	0.22 (0.15; 0.34) (*n* = 37)	0.007
Lactoferrin (ng/mL)	307.0 (254.5; 537.5)	331.0 (240.0; 659.0) (*n* = 43)	0.81	337.5 (256.3; 494.0) (*n* = 40)	234.0 (180.0; 376.0) (*n* = 43)	0.017
Lactoferrin/Neutrophil (pg)	43.7 (30.6; 68.7) (*n* = 42)	35.4 (24.2; 78.7) (*n* = 40)	0.40	70.9 (42.3; 106.2) (*n* = 39)	66.2 (47.5; 111.3) (*n* = 41)	0.39

*n*, number of cases; PE, Preeclampsia; UCB, umbilical cord blood.
